# Spreading Depolarization as a Therapeutic Target in Severe Ischemic Stroke: Physiological and Pharmacological Strategies

**DOI:** 10.3390/jpm12091447

**Published:** 2022-09-01

**Authors:** Lily Chau, Herbert T. Davis, Thomas Jones, Diana Greene-Chandos, Michel Torbey, C. William Shuttleworth, Andrew P. Carlson

**Affiliations:** 1 Department of Neurology, University of New Mexico, Albuquerque, NM 87131, USA; 2 Department of Internal Medicine, University of New Mexico, Albuquerque, NM 87131, USA; 3 Department of Psychiatry, University of New Mexico, Albuquerque, NM 87131, USA; 4 Department of Neuroscience, University of New Mexico, Albuquerque, NM 87131, USA; 5 Department of Neurosurgery, University of New Mexico, Albuquerque, NM 87131, USA

**Keywords:** spreading depolarization, ischemic stroke, edema progression, cerebral autoregulation

## Abstract

Background: Spreading depolarization (SD) occurs nearly ubiquitously in malignant hemispheric stroke (MHS) and is strongly implicated in edema progression and lesion expansion. Due to this high burden of SD after infarct, it is of great interest whether SD in MHS patients can be mitigated by physiologic or pharmacologic means and whether this intervention improves clinical outcomes. Here we describe the association between physiological variables and risk of SD in MHS patients who had undergone decompressive craniectomy and present an initial case of using ketamine to target SD in MHS. Methods: We recorded SD using subdural electrodes and time-linked with continuous physiological recordings in five subjects. We assessed physiologic variables in time bins preceding SD compared to those with no SD. Results: Using multivariable logistic regression, we found that increased ETCO2 (OR 0.772, 95% CI 0.655–0.910) and DBP (OR 0.958, 95% CI 0.941–0.991) were protective against SD, while elevated temperature (OR 2.048, 95% CI 1.442–2.909) and WBC (OR 1.113, 95% CI 1.081–1.922) were associated with increased risk of SD. In a subject with recurrent SD, ketamine at a dose of 2 mg/kg/h was found to completely inhibit SD. Conclusion: Fluctuations in physiological variables can be associated with risk of SD after MHS. Ketamine was also found to completely inhibit SD in one subject. These data suggest that use of physiological optimization strategies and/or pharmacologic therapy could inhibit SD in MHS patients, and thereby limit edema and infarct progression. Clinical trials using individualized approaches to target this novel mechanism are warranted.

## 1. Introduction

Malignant hemispheric stroke (MHS) occurs when a large territory of infarction (most commonly MCA territory) results in progressive space-occupying cerebral edema that can lead to secondary ischemia, brainstem compression, herniation, and death (malignant edema) [1,2]. Medical strategies for MHS management are limited and focus on treating malignant edema and resultant increased intracranial pressure [3,4,5]. These approaches include sedation with general anesthetics, pharmacologic hyperosmotic therapy, and targeting general physiological parameters such as blood pressure, temperature and blood glucose [3,4,5]. Surgical management of malignant MCA stroke with decompressive hemicraniectomy significantly improves mortality and morbidity in young patients (between 18 and 60 years old) when decompression is performed within 48 hours of stroke onset [6,7,8]. Widespread acceptance of surgical intervention remains somewhat limited due to surgical morbidity and an increased risk of survival with severe disability [9,10]. In addition, there are variable patient and family perceptions of quality of life with severe deficits [11,12].

The source of malignant edema in MHS has been poorly defined; however, a recent elegant study used a murine model of stroke to demonstrate that flow from cerebrospinal fluid (CSF) (rather than from a cellular or osmotic source) is the dominant source of vasogenic edema [13]. Remarkably, the mechanism for CSF influx was found to occur during episodes of spreading depolarization (SD) propagating around the infarct core [13]. In the metabolically compromised ischemic penumbra, SD was associated with spreading ischemia [14] and vascular collapse, allowing influx of CSF into the perivascular space. In normally perfused brain, SD resulted in spreading hyperemia and no CSF influx and edema was noted [13].

SD is an electrophysiological phenomenon characterized by massive, slowly propagating ionic shifts resulting in near complete cellular depolarization and transient depression of spontaneous cortical activity [15]. While spreading depolarization was first identified in otherwise healthy brain tissue (originally termed spreading depression) [16], it was subsequently detected in the acutely injured brain, where it is associated with increased neuronal damage [17,18,19]. SD occurs nearly ubiquitously in human patients with malignant hemispheric stroke (MHS) monitored with gold standard subdural electrocorticographic (ECoG) recordings [20]. In addition to contributing to edema expansion as above, mounting evidence also supports a role of SD in secondary infarct growth, related to episodic excitotoxic release of glutamate [21] and spreading ischemia [14]. In addition, multiple preclinical stroke models demonstrate that blockade of SDs is associated with reduced infarct volumes [22,23,24]. Conversely, experimental induction of additional SDs in the ischemic hemisphere resulted in increased infarct volumes [25,26,27].

Given the potential clinical implications of SD propagation in edema formation and the expansion of ischemic injury, SD is an attractive therapeutic target for limiting infarct expansion and edema in MHS. Previous studies have shown associations between physiological variables and the risk of SD in acute brain injury [28,29]. Physiologic factors that result in “supply-demand mismatch” have been strongly implicated, particularly in traumatic brain injury, in increasing the risk of SD [28]. In addition, inhibition of SD with ketamine has been clearly documented in aneurysmal subarachnoid hemorrhage and traumatic brain injury [30,31,32,33]. Due to the severity of MHS, it has been unknown whether physiological variables and pharmacological targeting with ketamine or related agents would be feasible [34]. 

In the current study, we used high resolution multimodality monitoring data to determine whether spontaneous fluctuations in physiological factors are independently associated with SD in a preliminary group of MHS patients. In addition, we demonstrate suppression of recurrent SD in one subject with recurring clusters of SD. Such analysis could potentially provide guidance for studies of optimal physiological and pharmacological strategies to target SD in MHS, with the ultimate goal of limiting edema and ischemic expansion.

## 2. Materials and Methods

### 2.1. Patients

Ten patients with large territory strokes with malignant edema who underwent decompressive craniectomy were recruited for the study. Consent was obtained from a legally authorized representative for all patients under prospective IRB approved protocols (UNM HRPO 10-159 and 17-297). During electrocorticographic (ECoG) monitoring time, patients received routine clinical care by neuro-intensivists [3]. 

### 2.2. Electrocorticographic Monitoring

Monitoring was performed using the standard previously published approach [35]. After completion of the decompression including durotomy, a standard 1 × 6 platinum cortical electrode (Integra Epilepsy) was placed in a visually estimated peri-infarct region. The lead wire of the strip was tunneled subcutaneously. Monitoring was conducted using a full-spectrum direct current (DC) amplifier and collected using a Moberg Component Neuromonitoring system (CNS; Moberg Research Inc., Ambler, PA, USA). Collected ECoG data was exported to LabChart (version 8.0, ADInstruments, Colorado Springs, CO, USA) where raw DC data for each channel as well as high-frequency filtered (0.5–50 Hz) data were displayed, together with the 60-second integral of the power of the filtered data, as per standard analysis and scoring recommendations [35].

### 2.3. Data Analysis

ECoG data was scored for SD and iso-electric SDs according to standard criteria [35]. For the purposes of this study, SD and isoelectric SD were grouped together for analysis. In addition to ECoG data, multiple continuous measures were collected in a time-locked fashion using the Moberg CNS. These included continuous peripheral oxygen saturation (SpO2), end tidal CO2 (ETCO2), mean arterial pressure (MAP), systolic blood pressure (SBP), diastolic blood pressure (DBP), and heart rate (HR). Additionally, hourly partial pressure of arterial oxygen (PaO2), partial pressure of carbon dioxide (PaCO2), temperature, white blood cell count (WBC), glucose, and lactate data were extracted from the electronic medical chart and time-synced to the continuous physiology data. We then binned data based on bins that ended in SD and bins that contained no SD. Exploratory sensitivity analysis was performed evaluating bin sizes of 5, 20, 30, and 60 min and showed similar results (data not shown); we chose 20-min bins for all analyses below.

### 2.4. Statistical Approaches

First, we assessed the probability of SD (bins ending in SD) across ranges of the available continuous variables. We only used continuous variables where there was data in >1 subject. Next, we sought to assess the risk factors for SD using logistic regression. We assessed the mean values for bins ending in SD compared to bins with no SD. Variables with intermittent measurements were carried forward in the data set until there was a different value entered. Missing data were imputed using SAS proc MI. We then incorporated factors that were significantly associated with SD (defined as 95% CI non-inclusive of 1.0) into a multivariable model to determine which risk factors were independently associated with SD. Analysis was conducted using MATLAB v2021b and SAS 9.4 (Cary, NC, USA). 

## 3. Results

### 3.1. Recordings

SD was detected only in ECoG recordings of all patients where electrodes were placed in viable tissue (five patients) (Table 1). For the other five patients, recordings demonstrated isoelectric or near isoelectric activity, consistent with placement within the infarct core and limiting the ability to detect SD. These observations are consistent with a prior report of SD in MHS [20]. All patients had large hemispheric stroke of ischemic origin secondary to large vessel occlusion, except subject 2, who had multifocal lobar hemorrhagic strokes secondary to infective endocarditis. There were no adverse events related to electrode strip placement or monitoring.

### 3.2. Physiological Variables Affect the Probability of SD

We performed exploratory sensitivity analysis to determine the optimum time window preceding SD and found similar trends for 5-, 20-, 30-, and 60-min bins (data not shown). We chose 20 min as a plausible latency that would capture all SD triggered in response to physiological changes without overlapping the time period of slow SD propagation, during which SD re-initiation cannot occur.

**Figure 1:**
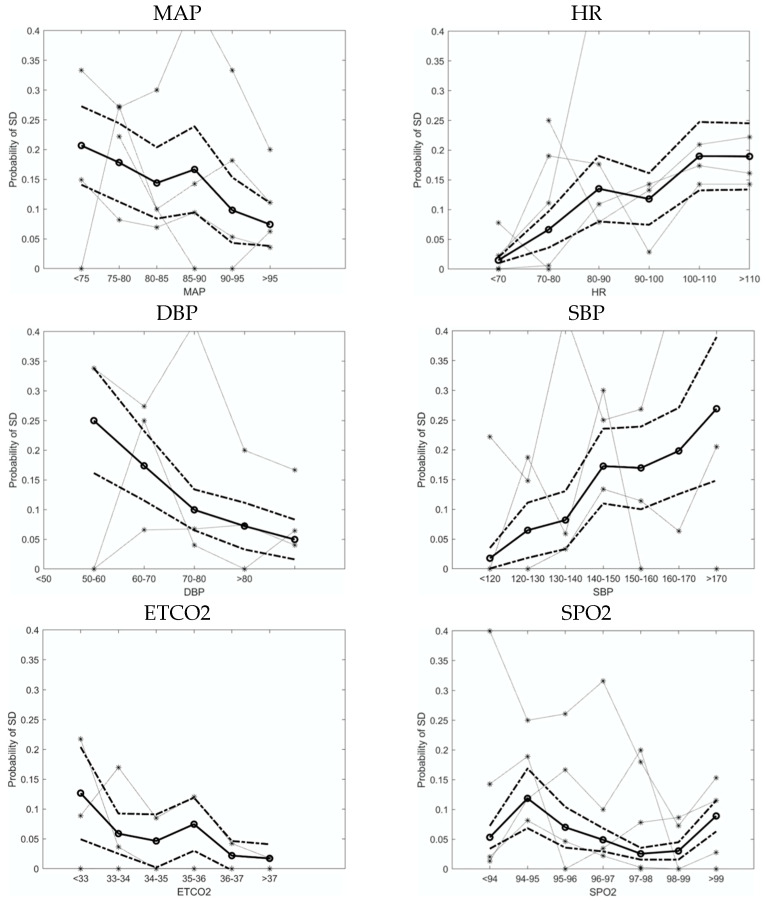
Probability of SD as a function of 20-min time-binned continuous variables, with probability curves of individual patients shown separately. The bold lines represent the mean and 95% CI probability curves for all patients. Lower MAP was associated with increased probability of SD and the effect appears to be driven by lower DBP. Lower ETCO2 and higher HR was also associated with increased probability of SD. MAP = mean arterial pressure, HR = heart rate, DBP = diastolic blood pressure, SBP = systolic blood pressure, ETCO2 = end tidal CO2, SPO2 = peripheral oxygen saturation.

Plotting the probability of SD in 20-min bins, we identified blood pressure, heart rate, ETCO2 and oxygen saturation for analysis (Figure 1). There was much interpatient variability in the data but the mean probability curves revealed some distinct trends. We identified a nearly linear relationship of lower MAP with increased probability of SD. The trend that we and others have noted in previous data sets of patients with aneurysmal subarachnoid hemorrhage [36] and traumatic brain injury [28], where probability of SD remains flat over the middle MAP range with an inflection point hypothesized to be related to autoregulatory failure, was not observed. Interestingly, such an inflection point was noted with diastolic blood pressure, but with an inflection point around 70 mmHg. Lower ETCO2 and higher heart rate also appeared to be associated with increased risk of SD. 

### 3.3. Risk Factors and Protective Factors for SD

To determine risk factors for SD, we performed univariate logistic regression using 20-min binned data for each physiological variable, defined as bins ending in SD compared to bins containing no SD. The univariable analysis consisted of the performance of a logistic regression on each variable independently to determine which variable to include in the multivariable analysis. The variables were selected to have a p-value less than 5% by being outside the 95% confidence interval for the null hypothesis of no effect. On univariable analysis (Table 2A), we found that increased temperature (measured in degrees Celsius) was most strongly associated with SD, with a twofold increase in the odds of SD (OR 2.048, 95% CI 1.442–2.909), and WBC was also significantly positively associated (OR 1.113, 95% CI 1.081–1.922). We also found physiological variables that were protective against SD (i.e., higher values were associated with decreased risk of SD); the strongest factors were ETCO2 (OR 0.772, 95% CI 0.655–0.910), PaCO2 (OR 0.936, 95% CI 0.900–0.973), and DBP (OR 0.958, 95% CI 0.941–0.991) (Table 2A). 

We used multivariable logistic regression to further define the independent predictive strength of each significant univariate variable. We performed a multivariable logistic regression analysis on all of the significant variables from the univariable analysis. The strongest multivariable predictors were temperature (OR 1.621, 95% CI 1.593–1.649) and WBC (OR 1.041, 95% CI 1.037–1.044). The strongest multivariable protective factors were ETCO2 (OR 0.943, 95% CI 0.940–0.946), PaCO2 (OR 0.973, 95% CI 0.971–0.975), and DBP (OR 0.986, 95% CI 0.984–0.989) (Table 2B). Taken together, results from the probability curves in Figure 1 and multivariable analysis in Table 2 indicate that higher ETCO2 and DBP appears to be most protective against SD. 

### 3.4. Ketamine Exhibits Dose-Dependent Inhibition of SD

**Figure 2:**
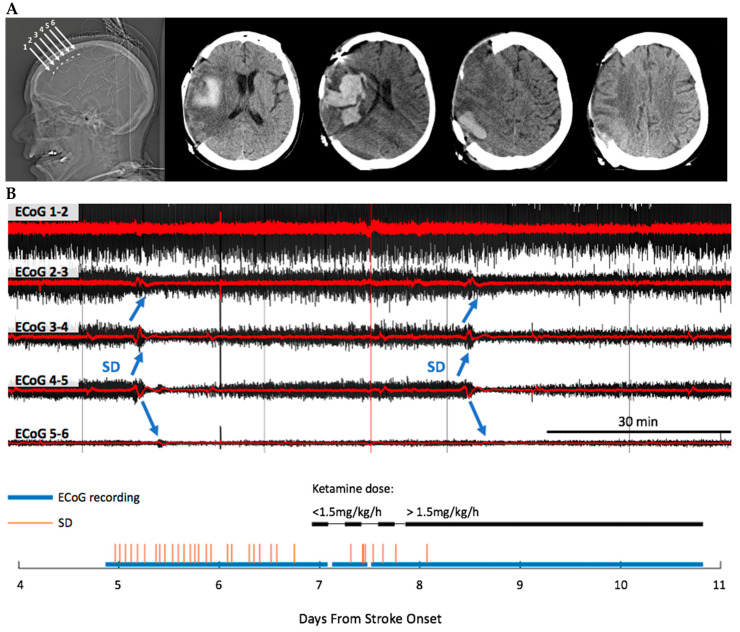
(**A**) MCA stroke with hemorrhagic conversion in subject 4. The electrode strip was placed in peri-infarct tissue radiating outward from ischemic region, such that electrodes 1 to 2 (corresponding to ECoG channel 1–2 in (**B**)) were located in the most normally perfused tissue, furthest from the infarct core, while electrodes 5 to 6 (corresponding to ECoG channel 5–6 in (**B**)) were located in the most hypo-perfused tissue, just bordering the infarct core. (**B**) A representative SD propagating across electrodes on the strip. A sequential bipolar montage of the five ECoG channels acquired from the electrode strip is depicted. The raw ECoG of a slow potential change spreading from ECoG channel 1–2 through sequential channels to ECoG channel 5–6 (shown in red) is associated with depression of ECoG activity (high-pass filtered ECoG (>0.5 Hz), shown in black). Bottom panel shows the total SDs observed during monitoring. Ketamine was initiated to treat intractable SDs associated with clinical decline. Only one SD occurred after ketamine dose was adjusted to therapeutic dose.

Intractable SDs were seen in subject 4 and during that time there was noted clinical decline with decreased level of consciousness. Because of these clinical changes in the context of multiple SD, a clinical decision was made to treat the patient with ketamine based on previously published data [30,31,32,33]. We found that SDs were essentially completely inhibited by therapeutic ketamine in a dose-dependent fashion. As a serendipitous internal control, ketamine was initially incorrectly dosed at 0.5 mg/h instead of the intended 0.5 mg/kg/h and no SD suppression was observed. The next day, the dosing issue was resolved and brought to a rate of 2 mg/kg/h, after which point only one isolated SD was noted during the remainder of the monitoring period (Figure 2). The dose of ketamine was then adjusted for clinical sedation between 1.5 and 3mg/kg/h; however there were no further SD at any of these doses. This dose-dependent effect of ketamine on SD is consistent with our previous study showing the effect of ketamine on SD in patients with traumatic brain injury and subarachnoid hemorrhage, where there was a threshold of no effect at doses lower than 0.55 mg/kg/h [32], a dose more than 20-fold higher than our initial incorrect dose. As expected with treatment of SD, ECoG power also improved after ketamine administration. Prior to administration of ketamine, ECoG power adjacent to the infarct core was substantially lower than in remote tissue. After the start of ketamine, ECoG power in peri-infarct tissue improved to a level similar to that in remote tissue (Supplementary Figure S1).

The treatment of SD appears to have resulted in improved clinical outcome, with regards to mortality and likely also morbidity, given the level of clinical decline at the time of initiation of treatment. At last clinic follow-up, about 2 months prior to the drafting of this manuscript, the patient was doing well, able to communicate and interact with family, as well as feed independently. However, the patient continued to experience post-stroke motor deficits requiring wheelchair assistance, as well as assistance for activities of daily living, with a modified Rankin scale score of 4. 

## 4. Discussion

In the context of metabolic compromise, SD appears to be triggered by imbalance in metabolic supply and demand [28,36,37,38]. In turn, the effect of SD on compromised tissue appears to be deleterious based on mounting strong mechanistic and outcome data [15,21,22,23,24,25,26,27,39,40,41,42,43,44]. In normal brain tissue, SD waves only cause transient dysfunction owing to intact neurovascular coupling, which mediates increased regional cerebral blood flow (CBF) to match increased ATP demand and restore membrane potential gradients across Na^+^/K^+^ATPase pumps (the hyperemic CBF response or spreading hyperemia), thus allowing for tissue recovery. However, in the injured brain, SD propagation can lead to further tissue injury due to impaired neurovascular coupling with reduction in regional CBF and tissue hypoxia (spreading ischemia), as well as SD-induced metabolite release, notably K^+^ and glutamate, causing further SD propagation and excitotoxicity, respectively. Therefore, while recoverable in healthy tissue, SD initiation in injured tissue can result in a vicious cycle of further injury and neuronal cell death [21,37,39,40,41,42].

It has, therefore, been a high priority to determine physiologic risk factors for SD so that critical care interventions could potentially be targeted to decrease the burden of such events [20,45,46]. In MHS patients, successive SDs have been associated with progressively worsening metabolic status with impairment of compensatory CBF responses, leading to increasing time to recovery of electrocorticographic (ECoG) activity [20,29,40,46]. Progressive tissue injury and metabolic compromise eventually leads to no recovery of ECoG activity, the EEG signature of ischemic expansion [44]. Due to the severity of the condition and other factors, there has been some nihilism concerning the role of aggressive SD targeted therapies in such a context [34]. However, given that decompressive craniectomy is required as a life-saving procedure in many of these patients, even limiting malignant edema formation and infarct progression through SD targeting approaches would be of benefit if some decompressive craniectomies could be avoided. Furthermore, if this process could be targeted, it could potentially be beneficial in smaller lesions where expansion could be more disabling.

Our observations add to the literature both by providing higher resolution averaged values as well as alignment of physiological variables to the time of SD onset in the injured brain. In addition, we present a multivariable analysis of such risk factors. Amongst the continuous variables we assessed, the probability of SD increased with decreasing MAP, DBP, and ETCO2. When both continuous and hourly recorded physiological variables were examined in a multivariable regression, we found that the strongest risk factors for SD were temperature and WBC, and the strongest protective factors were ETCO2 and PaCO2. These results are consistent with a previous study in patients with traumatic brain injury, where the probability of SD increased with decreasing MAP and increasing temperature [28], as well as a recent study in MHS patients which showed SD occurrence was preceded by a decrease in MAP over several hours [29]. However, our data adds temporal resolution as well as additional risk factors in a multivariable analysis. 

Our findings of the effects of MAP, DBP, and CO2 are important in relation to the mechanism of SD initiation, where supply–demand mismatch and decreased regional CBF leads to harmful SD initiation. The observation that a decrease in MAP precedes SD is perhaps surprising given that most of the increased risk occurs in what is considered the autoregulatory range. This strongly supports the hypothesis that cerebral autoregulation is disturbed in these patients, similar to previous reports in traumatic brain injury (TBI) [28] and aneurysmal subarachnoid hemorrhage (aSAH) [36]. The association of SD with impaired autoregulation was explicitly assessed in our recent study of aSAH patients, where the risk of SD was found to be increased during times with impaired autoregulation as measured by pressure reactivity (PRx) and oxygen reactivity (ORx) [36]. Thus, the effect of decreased MAP preceding SD may be a marker of the effect of low CBF conditions, consistent with the known mechanism for SD propagation. We plan to address this in future MHS patients with autoregulation measurement. The stronger effect of DBP compared to SBP is interesting, but will need to be assessed in a larger cohort to determine if this represents a consistent observation.

We also found that increasing CO2 levels was independently protective against SD, whether measured by end tidal monitoring or arterial levels. The physiological relationship between CO2 and CBF could be relatively straightforward, as an increase in CO2 level is known to cause cerebral vasodilation and subsequent increase in CBF [47,48]. However, since ICP has long been one of the only measurement tools in TBI, CO2 modulation has long been used to decrease ICP. In many ways, this is counter intuitive unless the ICP is related to severe hyperemia, and indeed severe hypocapnia has been found to be harmful, inducing cerebral ischemia despite ICP reduction [49,50,51]. Our data and others support that a more nuanced approach to optimize cerebral physiology is needed and perhaps a higher ICP should be tolerated in some situations if it improves perfusion and decreases SD.

It is important to note the interpatient heterogeneity in the data. It will be an important priority for future investigations to determine if there are risk factors that affect patient susceptibility to the harmful effects of secondary SD from physiological fluctuations, such as time after stroke, infarct size, and the presence of hemorrhage. 

Finally, we also present proof of concept data where recurrent SD were aborted with ketamine in a patient in whom such recurrent SD was thought to be contributing to neurological decline. Based on the suspected harmful effects of SD and previous clinical studies showing the effect of ketamine on SD [30,31,32,33], we sought to clinically treat this patient with ketamine. Very low dose ketamine did not stop SD, but doses ranging from 1.5–3 mg/kg/h (titrated for clinical sedation) seemed to effectively abort the recurrent SD. Although limited to one subject, this strongly supports a casual effect of ketamine on SD inhibition, consistent with prior studies in both experimental models and humans [30,31,32,33,52,53].

Ketamine inhibits SD by targeting the pathophysiological mechanism underlying SD propagation, which involves the release of neurotransmitter glutamate and activation of N-methyl-d-aspartate (NMDA) glutamate receptors. Ketamine is an NMDA receptor antagonist. Multiple experimental models have shown that ketamine perturbs the initiation and propagation of SD by affecting threshold to SD initiation, speed and duration of SD propagation, as well as the magnitude of extracellular ion shifts during SD [53,54,55]. In contrast, blockade of non-NMDA glutamate receptors, e.g., AMPA receptors, does not affect SD propagation [56,57].

Ketamine was the agent of choice in this current study. However consideration should be given to other NMDA receptor antagonists such as memantine in conditions where the sedating effects of ketamine would not be desirable and/or feasible, for example, in non-intubated stroke patients. Memantine is used to treat patients with Alzheimer’s disease and has been shown to inhibit SD initiation in experimental models [58,59]. In a recent clinical study, memantine improved recovery from SD by reducing SD duration and level of ECoG suppression [60]. Thus memantine may be a suitable alternative and/or combinatorial agent in cases where ketamine usage may be limited.

## 5. Conclusions

Taken together these results demonstrate that the risk of SD seems to be influenced by physiological fluctuations in MHS and that targeting physiological parameters with or without pharmacological intervention may be a reasonable strategy in MHS. Such a clinical trial using physiological and pharmacological targeting of SD is already underway in TBI (NCT#05337618). Future studies should expand on the most important physiological risk factors for SD in MHS. Clinical trials using such SD-linked targets in MHS are warranted and have the potential to not only limit infarct expansion but also reduce the need for decompressive craniectomy. 

## Figures and Tables

**Table 1:** Patient demographics.

Subject	Age	Sex	Stroke Territory	Comorbidities	Time to Craniectomy *	Duration of ECoG Monitoring	Total SD	Early mRS **
1	29	F	Left MCA, left PCA	T2DM, Alcohol use	27 h	96 h	40	4
2	24	M	Multifocal hemorrhagic	Polysubstance use	47 h	149 h	104	4
3	63	F	Right MCA	Unknown	69 h	74 h	20	4
4	64	F	Right MCA	TIA, HTN, HLD, CD	98 h	234 h	33	4
5	60	M	Right ICA terminus	HTN, Hyperthyroid	6 h	91 h	7	4

* Calculated from last known well time to time of craniectomy. ** Time of mRS at earliest clinic follow-up, ranging from 6 weeks to 3 months after discharge. mRS = modified Rankin Scale, T2DM = type 2 diabetes, TIA = transient ischemic attack, HTN = hypertension, HLD = hyperlipidemia, CD = cardiac disease.

**Table 2:** (**A**) Univariate logistic regression and (**B**) multivariable logistic regression with variables sorted by predictive strength.

	A. Univariable Analysis			B. Multivariable Analysis		
	Odds Ratio	95% Confidence Interval		Odds Ratio	95%Confidence Interval	
**Temp**	**2.048**	**1.442**	**2.909**	**1.621**	**1.593**	**1.649**
**WBC**	**1.113**	**1.081**	**1.922**	**1.041**	**1.037**	**1.044**
**HR**	**1.021**	**1.013**	**1.03**	**1.010**	**1.009**	**1.011**
**SBP**	**1.015**	**1.003**	**1.027**	*1.001*	*1.000*	*1.001*
**Glucose**	**1.013**	**1.003**	**1.022**	**1.014**	**1.013**	**1.014**
**PaO2**	**0.993**	**0.988**	**0.998**	*1.001*	*1.000*	*1.001*
**SpO2**	*0.986*	*0.921*	*1.055*	--		
**MAP**	**0.975**	**0.96**	**0.991**	**0.975**	**0.960**	**0.991**
**DBP**	**0.958**	**0.941**	**0.991**	**0.986**	**0.984**	**0.989**
**PaCO2**	**0.936**	**0.9**	**0.973**	**0.973**	**0.971**	**0.975**
**ETCO2**	**0.772**	**0.655**	**0.91**	**0.943**	**0.940**	**0.946**
**Lactate**	*0.644*	*0.398*	*1.042*	--		

In the multivariable analysis, the strongest predictors of SD were temperature (OR 1.621, 95% CI 1.593–1.649) and WBC (OR 1.041, 95% CI 1.037–1.044), while the strongest protective factors against SD were ETCO2 (OR 0.943, 95% CI 0.940–0.946), PaCO2 (OR 0.973, 95% CI 0.971–0.975), and DBP (OR 0.986, 95% CI 0.984–0.989).

## Data Availability

Not applicable.

## Supplementary Materials

The following are available online at https://www.mdpi.com/article/10.3390/jpm12091447/s1, Figure S1: Effect of ketamine on the power of ECoG.
