# Supplementary material for: Spreading Depolarization as a Therapeutic Target in Severe Ischemic Stroke: Physiological and Pharmacological Strategies
Source: J Pers Med. 2022 Sep 1;12(9):1447. doi: 10.3390/jpm12091447 (PMC9502975; doi:10.3390/jpm12091447)
Supplement: Supplementary file 1 [file jpm-12-01447-s001.zip › jpm-1788224-supplementary.pdf]

Supplementary Materials

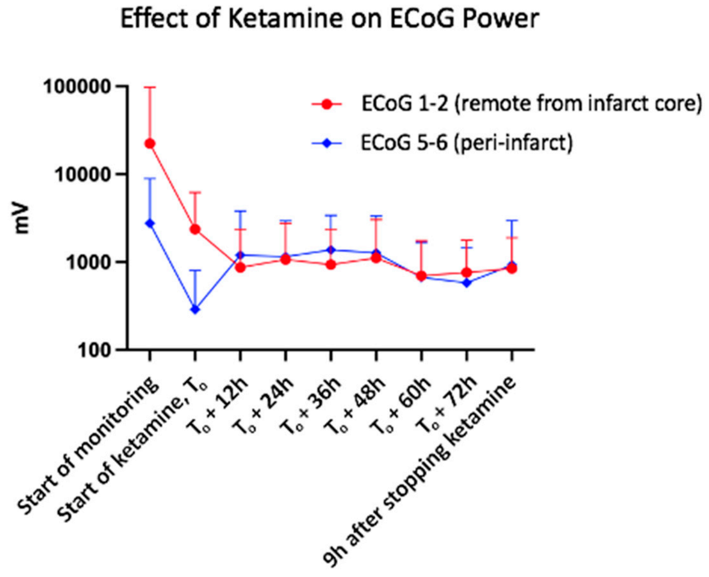

Figure S1. Effect of ketamine on the power of ECoG. Prior to administration of ketamine, ECoG power in peri-infarct tissue is substantially lower than that in remote tissue. After the start of ketamine, there is improvement in ECoG power in the peri-infarct region to a level similar to that of remote tissue. Data analysis modeled after previous studies [1,2]. .

Supplemental References:

1. Reinhart, K.M.; Shuttleworth, C.W. Ketamine reduces deleterious consequences of spreading depolarizations. *Exp. Neurol.* **2018**, *305*, 121–128. https://doi.org/10.1016/j.expneurol.2018.04.007.
2. Reinhart, K.M.; Humphrey, A.; Brennan, K.C.; Carlson, A.P.; Shuttleworth, C.W. Memantine Improves Recovery after Spreading Depolarization in Brain Slices and can be Considered for Future Clinical Trials. *Neurocrit. Care* **2021**, *35* (Suppl. 2), 135–145. https://doi.org/10.1007/s12028-021-01351-9.
